# Biomass-Derived Porous Carbon Materials for Supercapacitor

**DOI:** 10.3389/fchem.2019.00274

**Published:** 2019-04-24

**Authors:** Hui Yang, Shewen Ye, Jiaming Zhou, Tongxiang Liang

**Affiliations:** School of Materials Science and Engineering, Jiangxi University of Science and Technology, Ganzhou, China

**Keywords:** biomass, porous carbon, supercapacitor, electrochemical performance, energy storage

## Abstract

The fast consumption of fossil energy accompanied by the ever-worsening environment urge the development of a clean and novel energy storage system. As one of the most promising candidates, the supercapacitor owns unique advantages, and numerous electrodes materials have been exploited. Hence, biomass-derived porous carbon materials (BDPCs), at low cost, abundant and sustainable, with adjustable dimension, superb electrical conductivity, satisfactory specific surface area (SSA) and superior electrochemical stability have been attracting intense attention and highly trusted to be a capable candidate for supercapacitors. This review will highlight the recent lab-scale methods for preparing BDPCs, and analyze their effects on BDPCs' microstructure, electrical conductivity, chemical composition and electrochemical properties. Future research trends in this field also will be provided.

## Introduction

The ever-growing population, crisis of energy shortage, and environmental pollution caused by burning fossil fuels have brought about different kinds of problems, and they stimulate the process of developing clean and sustainable energy, such as solar energy, wind power etc. (Dunn et al., 2011; Chu and Majumdar, 2012; Dubal et al., 2015; Kamat, 2015; Kazmerski, 2016; Peng et al., 2017). All these closely relate to the development of the advanced energy storage system. Until now, for electrochemical energy storage systems, for example, various rechargeable batteries and supercapacitorsare identified as the most promising energy storage devices (Chmiola et al., 2006; Miller and Simon, 2008; Kim et al., 2010; Cheng et al., 2011; Xu et al., 2014; Gogotsi, 2015). Although batteries have a relatively longer life cycle, (< 1,000 cycles), high-energy density and satisfactory rate performance, distinct from rechargeable batteries, supercapacitors possess the following characteristics: high-power energy, extremely long cycle life (>100,000 cycles) without losing capacitance and safety, it is quite essential to bridge the gap between conventional electrochemical capacitors and rechargeable batteries, and meet the demand of electric vehicles which require high power (Liu et al., 2018a; Xu et al., 2018).

Basically, supercapacitors can be classified under two categories according to their charge storage mechanism, which further affects the power density of supercapacitors. The electrical double layer (EDL) capacitance and pseudo-capacitance are quite different. For the former, charges are stored through ions' adsorption by electro-static interaction at the near-surface of active materials, where EDL forms; while the energy storage/release mechanism for pseudo-capacitance is by means of redox reaction, ions insertion/extrusion and under-potential depositions (Augustyn et al., 2014; Mccloskey, 2015). Both the aforementioned supercapacitors require chemical and structure stability, outstanding electrical conductivity, and high SSA. Hence, to satisfy the requirement of high capacity and superior rate performance for supercapacitors, the exploitation of advanced electrode materials with low cost, adjustable composition, and microstructure (especially pore structure) turns increasingly important.

In the past decades, thanks to the excellent conductivity property, high SSA, variable pore structure and porosity, carbon materials, such as commercial activated carbons (Chan et al., 2004; Jänes et al., 2007; Zhang and Zhao, 2009; Lv et al., 2011; Wang et al., 2014), graphene (Wang et al., 2009; Liu et al., 2010; Zhang et al., 2010; Cao et al., 2011), graphene oxide (GO) (Li et al., 2013; Li and Yang, 2014; Down et al., 2018), carbon nanotubes (CNTs) (An et al., 2001; Futaba et al., 2006; Pushparaj et al., 2007), porous carbon (PC) (Lee et al., 2000; Vix-Guterl et al., 2005; Hou et al., 2015) and their composites (Salunkhe et al., 2015) have achieved various kinds of essential accomplishments on their volumetric performance. However, their high production cost, complicated and unsustainable preparation process greatly limited their wide application. Besides, the gravimetric capacitance for CAs is keeping at a relatively low level for several decades, that should be ascribed to the rich micro-pores, which are hard accessible for ions, especially at high-current density condition (Li et al., 2014). Therefore, enlarging the ion-accessible area through creating a suitable hierarchical porous structure is a key factor for achieving high specific capacitance (C_s_) and rate performance (Zhao et al., 2016). Biomass materials abundantly present across our surroundings, and their low cost, accessible, environmentally friendly, and recyclable properties ensure they are ideal candidates for resources of carbon materials. At the same time, their naturally hierarchical porous structure and various elements (N, S) facilitate electrolyte penetration and extra active sites' generation, respectively (Chen et al., 2016).

In this review, we provide a summary of the recent and significant advances in hierarchical porous carbon (HPC) derived from biomass materials, emphasizing on the relationship between interconnectivity of the pore structure and electrochemical efficiency, and synthetic strategies for preparing HPC as supercapacitors. Finally, the current challenges and future directions are briefly discussed.

## Relation Between Pore Features and Capacities

In general, the capacitance of PC materials relies on the pore size distribution, connectivity and hydrophilicity; these factors will be further influencing the ion diffusion process and energy density of active materials. According to the findings by Dubinin (1960) and the classification by the International Union of Pure and Applied Chemistry (IUPAC) in 1985 (Sing, 1985), pores with different widths can be divided among three categories: macropores (>50 nm), mesopores (2–50 nm) and micropores (< 2 nm). With the development of porous materials, three new pore types have been classified since 2015, they are nanopores (< 100 nm), supermicropores (0.7–2 nm) and ultramicropores (< 0.7 nm).

### Pore Size Distribution

**Figure 1:**
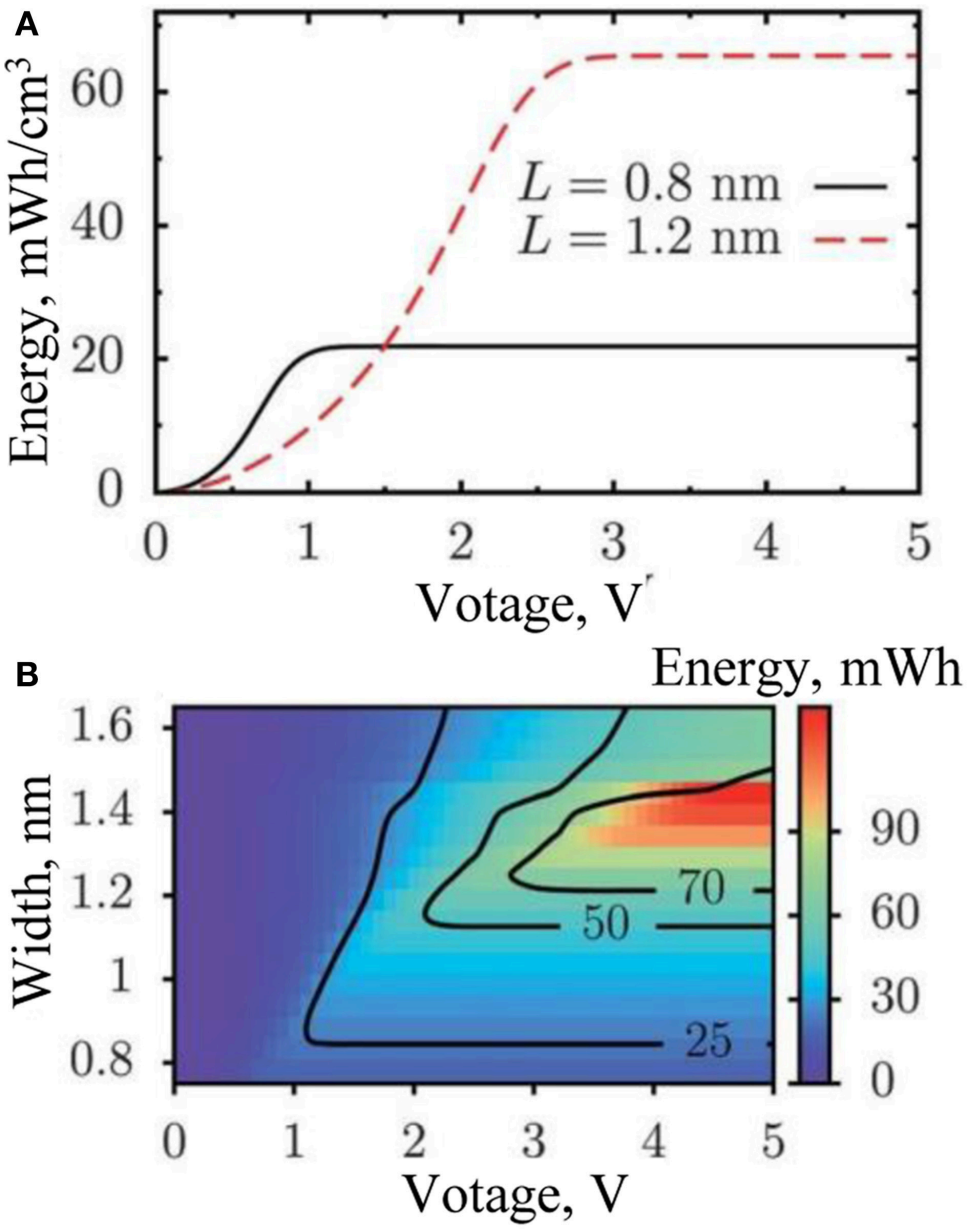
**(A)** The relationship between energy density and working voltage in PC supercapacitor. **(B)** The energy density correlates with pore size and working voltage. Reprinted with permission from Kondrat et al. (2012). Copyright 2012 Royal Society of Chemistry.

Although intense efforts are focused on improving the surface area and redox active sites of PC materials, optimization of pore size distribution is also an effective mode to enhance capacitance, especially for EDL capacitors. Generally, nanoporous carbons consist of pores ranging from micropores to mesopores, and most of them cannot maintain long-range ordering, or be accessible to ions from the electrolyte, though we know the electrochemical performance of PC is dependent on the interface of carbon and electrolyte. The macropore, mesopore and micropore have different functions in the electrochemical charge/discharge process. Macropores serve as ion-buffing reservoirs for meso- and micropores; mesopores provide abundant transport channels for ions' diffusion; in spite of some regions of micropores are inaccessible to adsorb ions, it still affects the charge status through controlling the diffusion of ions and molecular sieve effects, and then changing the capacitance of PC materials (Liu et al., 2018c); in addition, as the size of pores decreases toward <1 nm, an anomalous capacitance increase occurred in the organic electrolyte due to ion dissolution. Chmiola and co-workers reported that PC could achieve the highest capacitance as its average pore size matched the size of desolvated ions (Chmiola et al., 2006). Kondrat (Kondrat et al., 2012) found that different carbon materials with the same average pore size will display very different capacitive properties, because of the difference in pore size distribution; besides, for the monodisperse porous electrode, its energy density and pore size do not fit a non-monotonic function (Figure 1). Moreover, Cheng (Wang et al., 2008) believed that high micropore volume and the micropore-to-total-pore ratio are crucial for gaining a high-rate electrochemical performance of PC.

### Pore Connectivity

interconnected pores at different size dimensions are qualified as a hierarchical porous structure, which facilitates electrolyte infiltration and ion diffusion through a different pore canal (Borchardt et al., 2013). Until now, it is still a big challenge to construct carbon materials with 3D-interconnected, long-range order macroporous and mesoporous structures. These structures, combined with decent SSA and proper pore size distribution, allow efficient diffusion of any substance, such as electrolytes, ions etc., to the interior space of all channels. The strategies for fabrication HPC mainly includes templating (hard/soft) (Yuan et al., 2016; Xiong et al., 2017; Zhu et al., 2017) and non-templating ways (Lv et al., 2012), while these methods are time-consuming and highly costly. As a promising renewable resource, Biomass always possess a naturally interconnected, multichannel and porous structure, hence, they are the excellent candidates for preparing HPC. Wang and co-workers used pomelo peel as the carbon sources, in virtue of its foamy fibrous layer and abundant oxygen-containing functional groups, HPC was obtained after KOH activation, it gained a capacitance of 222.6 F/g at relatively low discharge current density (0.5 A/g), at the same time, it maintained a good rate of performance and a suitable cycling stability (Li et al., 2017a).

### Pores' Hydrophilicity

**Figure 2:**
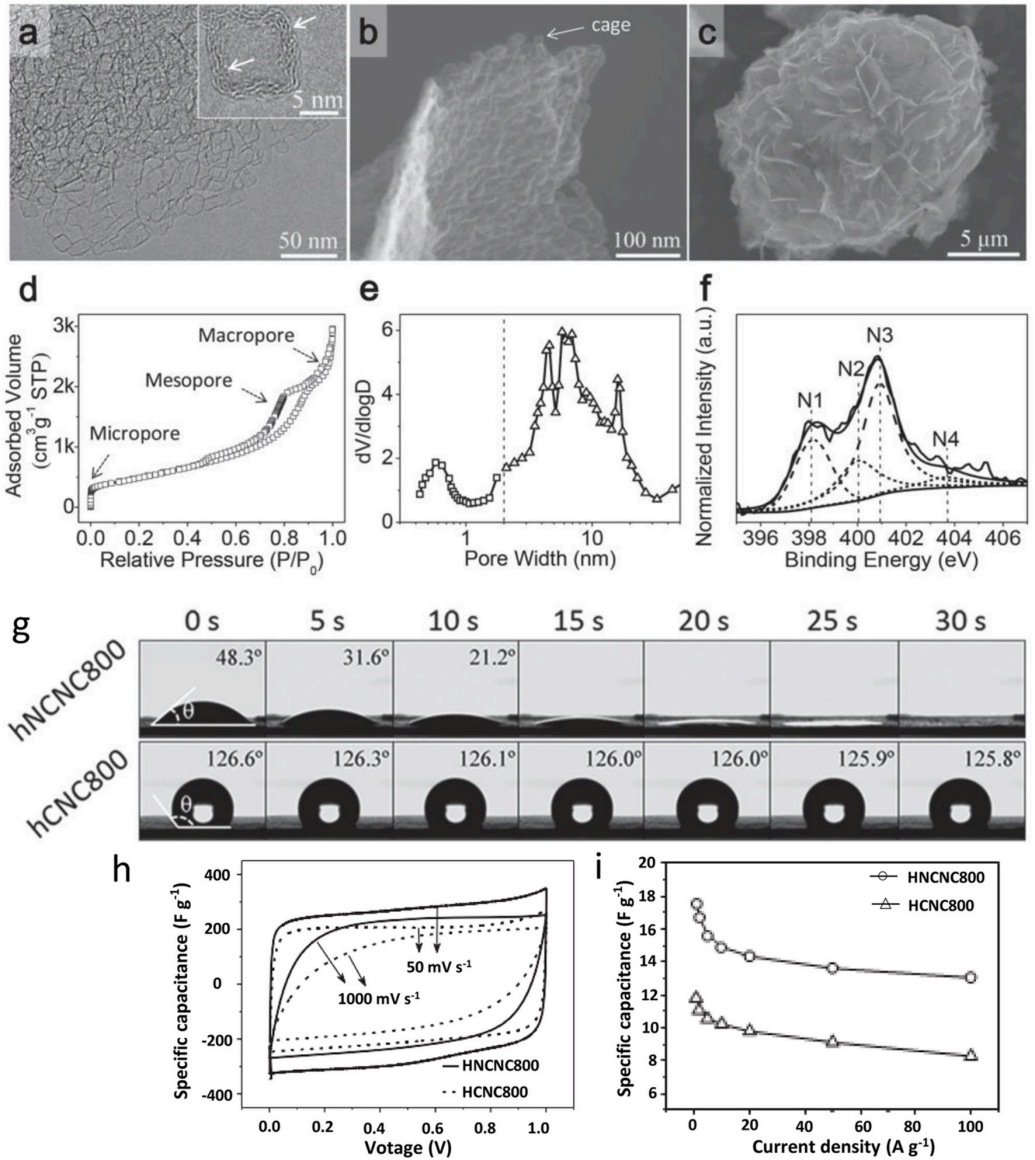
**(a–c)** TEM and SEM images of N-doped PC; **(d-f)** N_2_ adsorption/desorption isothermal, pore size distribution and XPS spectra of the sample; **(g)** wettability tests; **(h,i)** electrochemical performance analysis. Reprinted with permission from Zhao et al. (2015). Copyright 2015 Willey VCH.

Until recently, the charging mechanism for supercapacitors is derived from ions into the PC network by applied potential driving. Hence, the pore wettability, also named as surface hydrophobic/hydrophilic balance, closely relates to pores' surface functional groups, and has a great impact on the penetration of guest species into the pore systems and transfer of electrons (Xiao et al., 2016). Therefore, the functionalization of PC materials through surface modification gradually becomes an important issue to achieve appropriate wettability (Figure 2). The modification techniques always involve post-treatment of PC materials in oxidizing media, or doping carbons with oxygen, nitrogen and other elements (Lin et al., 2015; Chen et al., 2017b). Generally, N- and O-doping, introduced by grafting different functional groups, facilitate adsorption of ions and further improve the hydrophilicity of the carbon matrix. Therefore, moderate oxidation accompanied by heteroatom doping provide faradic pseudocapacitance, and then increase the capacitance value of electrode materials. However, overoxidation results in collapse of pore structure and large interface resistance (Wei et al., 2016). Moreover, most natural biomass contains nitrogen, boron, sulfur and other trace elements, which would be doped into the carbon framework as heteroatoms, and that will generate a more active site, and decrease the hydrophobicity of PC. Li utilized corncob as a precursor, and obtained nitrogen-doped activated carbons with N content up to 4 wt%, the corresponding hybrid-type supercapacitor achieved high-energy density and rate performance (Li et al., 2015a).

## Brief Overview of Fabrication Strategies for PC

Up to now, various synthetic methods have been developed for preparing biomass-derived PC. The carbonization methods, such as pyrolysis (Dubal et al., 2016) and hydrothermal carbonization (Enterría et al., 2016), are used earliest to obtain PC. The pyrolysis is a dry-carbonization reaction, which usually takes place in an inert atmosphere or low oxygen environment at an elevated temperature condition (300–900°C), the main components of biomass are gradually transferred into biochar through a series of reaction, such as cross-linking depolymerization, fragmentation reactions etc. The performance of biochar depends on the reaction temperature, time and catalyst (Li et al., 2016b); For the hydrothermal carbonization, it is carried out in aqueous environment at elevated temperature (< 300°C) and autogenous high pressure, it is a chemical process for conversion of biomass to carbonaceous materials, whose properties are determined by reaction temperature, time, pressure, and water/biomass ratio. Furthermore, Compared with the pyrolysis reaction, hydrothermal carbonization results in higher biochar yield. However, biochars obtained through the aforementioned methods have low SSA and porosity. Hence, as the most common strategies for increasing SSA of carbon materials, activation, including physical and chemical activation, have been wildly used.

Physical activation is carried out at a high temperature (>700°C) in the presence of gases like CO_2_, H_2_O, air and ozone (Abioye and Ani, 2015; Chang et al., 2016; Lota et al., 2016). The process of physical activation has two steps: firstly, carbonization happens at a low temperature in an inert atmosphere, and during this process, volatile matters are eliminated and biochar is formed. Subsequently, gasification reaction leads to the formation of abundant open pores because of the introduction of oxidizing gas at a high temperature. It should be noteworthy that increasing reaction temperature and prolonging treating time are helpful to improve the porosity of carbon materials, while the pore size distribution will be broadened.

**Figure 3:**
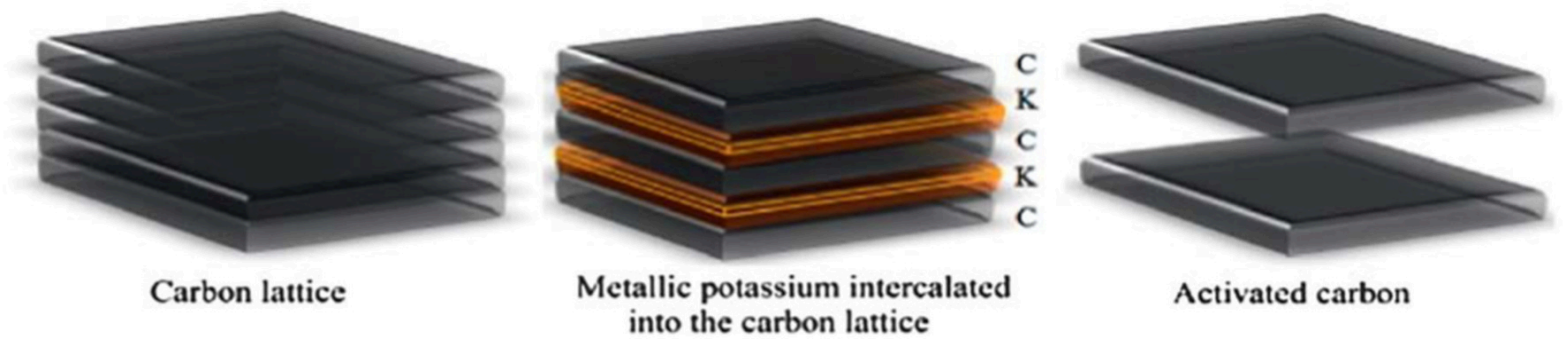
KOH activation mechanism. Reprinted with permission from Wang and Kaskel (2012). Copyright 2012 Royal Society of Chemistry.

Prior to carbonization, biochar is pre-mixed with certain chemicals, such as an acid (Sun et al., 2015), strong base (Qu et al., 2015), or a salt (Sevilla and Fuertes, 2016), then the mixture is carbonized at relatively low temperatures (450–900°C). Although there are drawbacks for chemical activation, for example, high cost, apparatus corrosion, and non-recoverable chemicals, it is still preferred over physical activation owing to its lower reaction temperature, shorter reaction time and larger SSA. Among various kinds of activating reagents, KOH (Lv et al., 2011) is the most wildly used chemical. The activation mechanism of KOH activation (Figure 3) can be summarized and shown as the following reaction equations.

(1) $$\begin{array}{l} KOH+C↔K+CO_{2}+H_{2}O \end{array}$$

(2) $$\begin{array}{l} KOH+CO_{2}↔K+CO_{2}+H_{2}O \end{array}$$

(3) $$\begin{array}{l} KOH+C↔K+H_{2}+K_{2}CO_{3} \end{array}$$

(4) $$\begin{array}{l} C+CO_{2}↔CO \end{array}$$

### Cellulose-Derived PC

As the most abundant and sustainable natural polymer, cellulose is the main component of green plants' primary cell wall and consists of a linear chain of D-glucose. Cellulose fibers exhibit high surface area and aspect ratios, excellent mechanics and flexibility, broad chemical-modification capacity, so it has attracted intense attention in the past several years for supercapacitor (Yang et al., 2015; Yu et al., 2016). Generally, according to the origin of cellulose, it can be classified in two ways: commercial cellulose and isolated cellulose from lignocellulose for PC precursor. After carbonization and activation through a physical or chemical process at elevated temperature, then PC was gained. Although they have extremely high SSA, compared with HPC, it exhibited limited C_s_ because of low wettability, slow ions' diffusion rate and relatively small amount of the effective active site. Hence, recently, many efforts have been focusing on the fabrication of HPC derived from cellulose.

**Figure 4:**
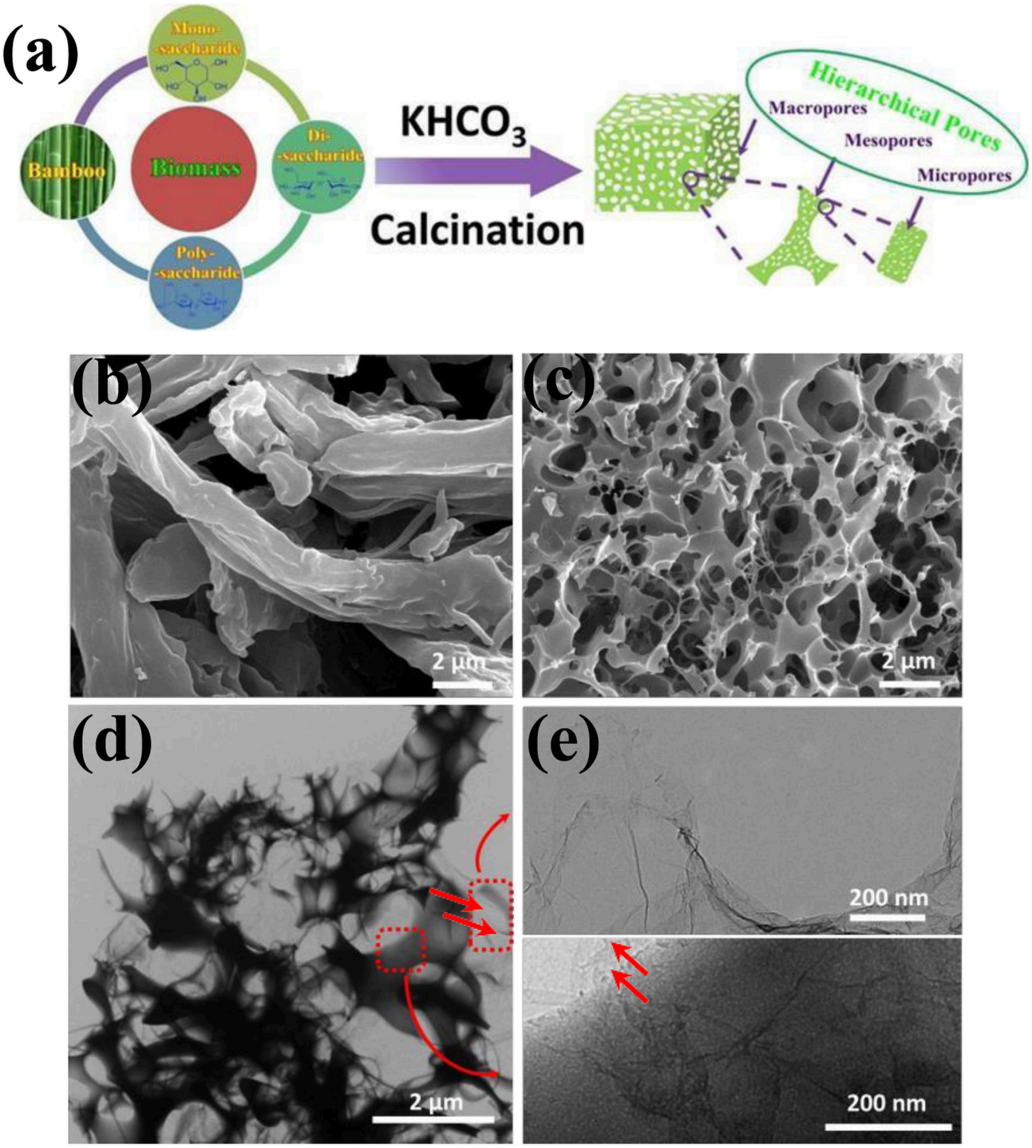
**(a)** Scheme diagram of the formation of HPC by “leavening” agent; **(b–e)** SEM and TEM images of HPC. Reprinted with permission from Deng et al. (2015). Copyright 2015 Royal Society of Chemistry.

The unique structure of carbon aerogels provides high SSA, hierarchical porous structure, interconnected macro-/meso-/micropores, efficient ions diffusion rate, and abundant active sites. Nanocellulose microcrystalline would be gelling at the base condition, after freeze-drying and CO_2_ activation process, carbon aerogel with interconnected 3D nanostructure could be prepared. The final products showed high SSA (1,873 m^2^/g) and high pore volume (2.65 cm^3^/g); besides, the C_s_ reached 302 F/g and 205 F/g at 0.5 A/g and 20 A/g, respectively (Zu et al., 2016). “leavening” strategy (Figure 4) is applicable to various biomass and their derivatives, the corresponding electrode materials exhibited a C_s_ of 253 F/g with superior cycling stability (Deng et al., 2015). Zhuo et al. synthesized HPC through a dissolving-gelling process, after carbonization process, carbon aerogel with abundant macropores, mesopores and micropores was obtained, and gained high C_s_ of 328 F/g at 0.5 A/g with 96% of the capacitance retention after 5,000 cycles (Zhuo et al., 2016). Sodium carboxymethyl cellulose aerogels derived PC could be obtained through sol-gel and KOH activation, its relative low C_s_ (152 F/g at 0.5 A/g) might be attributed to the limited SSA (<500 m^2^/g) (Yu et al., 2016). What's more, Long et al. cellulose nanofibrils and short cellulose nanofibrils were assembled into macro/micro/mesoporous structure by Li and co-workers, who found the hierarchical structure maintained high surface area (1,244 cm^2^/g), good C_s_ (170 F/g) at high current densities (Li et al., 2017b). Waste paper is also a promising candidate for preparing PC. Kraft pulp was mixed with KOH solution and freeze-dried, then the mixture was calcined at an elevated temperature under an inert atmosphere. Dense graphene-like HPC was fabricated, and its gravimetric and volumetric C_s_ is 309 F/g and 309 F/cm^3^ at 1 A/g, respectively (Mo et al., 2018).

It is believed that increasing surface functional groups into the carbon framework contribute to the enhancement of the capacitance of supercapacitors. Hence, introducing of N, B, P, and O, which act as electron donor or electron acceptor, have the following effects: improving wettability between PC and electrolyte; facilitate the binding between carbon materials and ions; enhancing the active sites in the carbon framework (Chen et al., 2013, 2017d; Wu et al., 2015; An et al., 2017). Contrary to oxygenic functional groups, N-containing functional groups own basic characters, which leads to donor-acceptor properties, and improves the final electrochemical properties of electrode materials (Wang et al., 2008). As the carbon sources and as a template, cellulose nanocrystals were used to control the growth of melamine-formaldehyde, then rod-like porous N-doped carbon particles were fabricated; they achieved 352 F/g at 5 A/g and maintained over 95% C_s_ retention after 2000 cycles (Wu et al., 2015). Through pyrolysis and activation procedure, PC nanosheets (PCN) were doped by N and S, the co-doped PCN had a C_s_ of 298 and 233 F/g at charge/discharge current density of 0.5 and 50 A/g. Besides, only 2% capacitance lost after 10,000 cycles (Li et al., 2016b). Phosphorus (P) doped HPC possessed outstanding rate capability, and it showed a C_s_ of 133 F/g (146 mF/cm^2^) at 10 A/g with ~98% capacitance retention after 10,000 cycles (Yi et al., 2017).

### Lignin-Derived PC

**Figure 5:**
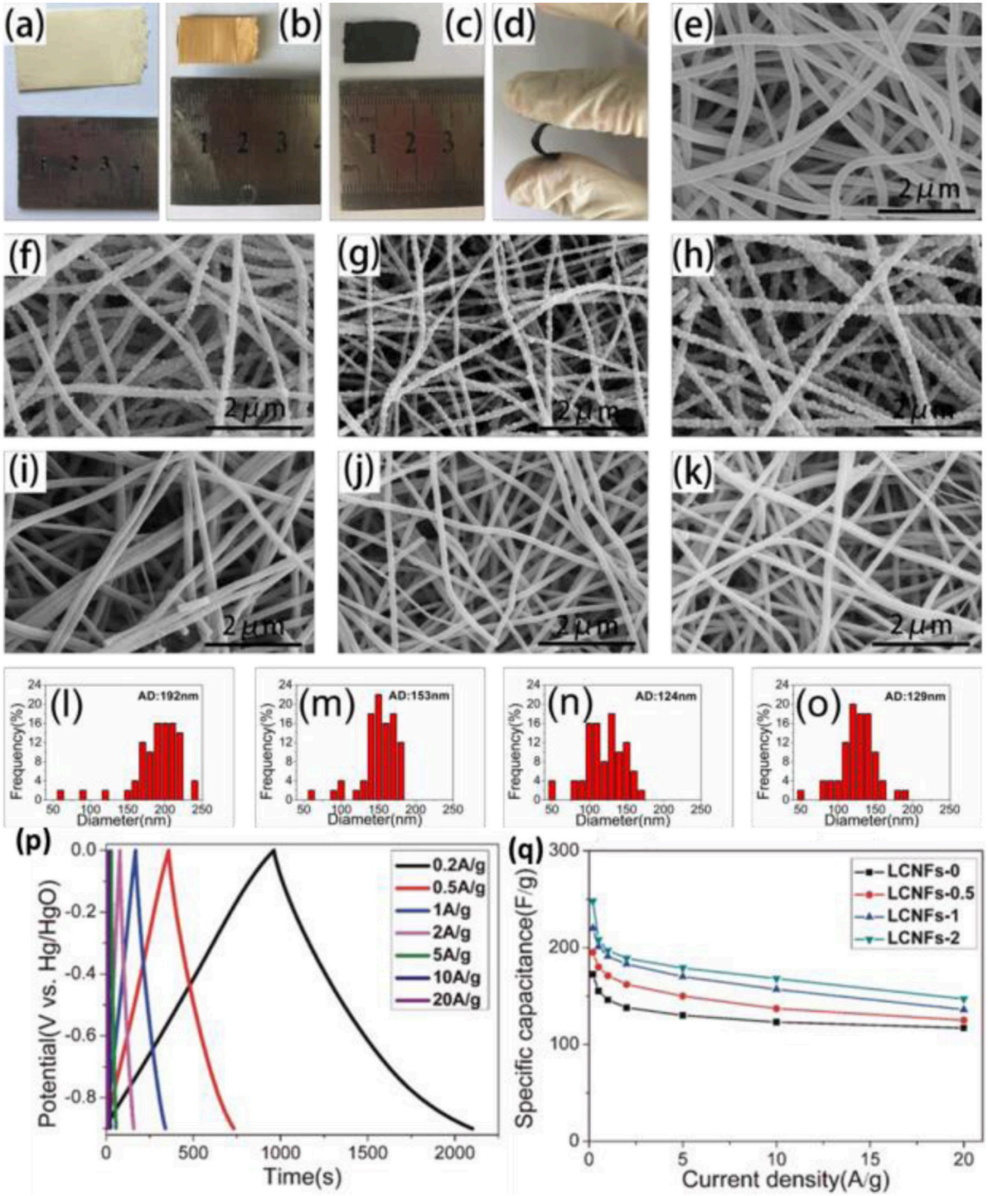
Optical images **(a–c)** and SEM images **(e–k)** of fiber film; **(l–o)** size distribution of carbon fiber; galvanostatic charge and discharge curves **(p)** and rate capability **(q)** of fiber film. Reprinted with permission from Ma et al. (2018). Copyright 2018 Elsevier.

As the most abundant natural aromatic polymer and the second raw material from plants, lignin, with a production ~ 50 million tons per year, is known as one of the most promising candidates for carbon sources considering environmental and economic aspects (Suhas and Carrott, 2007). Recently, the lignin-based PC nanocomposite gradually has attracted much attention because of its excellent electrochemical performance (Kai et al., 2016; Ma et al., 2016; Wang et al., 2016a). Generally, it is quite difficult to isolate the lignin because of the potential oxidation and condensation reaction during the isolation process; besides, the mass production and purity of lignin, isolated through hydrolysis or solubilization of plants, also limit the wide application of lignin-based PC for supercapacitor. However, series of works have been published and some progress has been made. Physical and chemical activations are the most common strategy to prepare lignin-based HPC. Zhang (Zhang et al., 2015a) utilized KOH as the template and activating agent and successfully prepared lignin-derived HPC with an interconnected 3D network. Its abundant oxygen-containing group ensures a relatively good pseudocapacitance performance (165 F/g in 1 M H_2_SO_4_ at 50 mA/g). Increasing SSA of carbon materials is a good choice for promoting the C_s_ of electrode materials; hence, a higher HPC derived from lignin was obtained by KOH activation, the corresponding products provided a C_s_ of 286.7 F/g at 0.2 A/g (Zhang et al., 2015b). In addition, a hard template, such as zeolite, was used to control the assembly of lignin, and then control the pore structure of the final products, this lignin-based templated PC showed a high C_s_ (250 F/g at 50 mA/g) (Ruiz-Rosas et al., 2014). One dimension of PC fibers derived from lignin not only contributed to faster electron conduction, but also facilitated the electrolyte infiltration by providing abundant pore structure, Hu et al. reported that porous fiber activated by KOH achieved a total capacitance of 1 F at 10 mg loading (Hu et al., 2014). Besides, lignin-based HPC film was developed by Chang and co-workers, as shown in Figure 5, a flexible film consists of lignin, PVP and Mg (NO_3_)_2_ was synthesized through electrospinning, then the precursor was carbonized and pickling to remove the MgO, the final PC film exhibited C_s_ of 248 F/g and outstanding rate and cyclic performance (Ma et al., 2018).

To enhance the performance of lignin-derived PC, surfactant, organic solvent and silica were added into the mixture of lignin, after carbonization and activation by 2 M NaOH, the carbon film (~310 μm) was used as an electrode, because of its hierarchical pore structure and superb electrical conductivity, this novel carbon electrode achieved an ultrahigh areal capacitance of 3 F/cm^2^ and a high volumetric capacitance of 97.1 F/cm^3^, high mass loading and excellent electrochemical performance ensure it is a promising candidate for supercapacitor (Li et al., 2016a). Furthermore, lignin-derived byproducts were used as carbon resources by hydrothermal treatment and activation, the as-prepared N-doped PC with hierarchical bowl-like pore structure exhibited a high conductivity (4.8 S/cm), favorable C_s_ (312 F/g at 1 A/g in 6 M KOH) and excellent rate capability (81% retention at 80 A/g) (Wang et al., 2017). Moreover, Wang reported that N-doped PC derived by KOH activated urea-modified lignin could be obtained; it possessed a well-developed porous structure and extremely high SSA (3,130 m^2^/g). The corresponding supercapacitors achieved C_s_ of 273 and 306 F/g in 6 M KOH and KOH-PVA solid electrolytes, respectively (Wang et al., 2016b).

### Alginate-Derived PC

Except for cellulose, other kinds of natural polysaccharides obtained by artificial extracting have been wildly used as PC precursor because of their low cost, accessibility and component stability.

Alginate is a polysaccharide composed by covalently linked mannuronate and guluronate, and widely distributed to the cell walls of brown algae. Furthermore, the chelation between metal ions and alginate results in gelling and forming an “egg-box” structure (Davis et al., 2003), which further being calcinated at 800°C, three-dimensional macro-meso-microporous HPC aerogels were prepared. The final products showed an excellent rate performance (65% capacity retention at 100 A/g) and decent capacity (188 F/g at 1 A/g) (Wang et al., 2018a). Li and co-workers synthesized alginate gels with interconnected macropore structure utilizing freeze-drying process, then after activation by KOH and removal of Ca, interconnected HPC was obtained; it showed a high-rate capability with 222 F/g and long cycling life at 10 A/g (Li et al., 2015c). On account of the limited C_s_ of an EDLC capacitor, O- and N- enriched PC was prepared by calcining kelp in NH_3_ atmosphere, the products acquired high volumetric C_s_ (>360 F/cm^3^) and superb cycling performance (Li et al., 2015b). In addition, Nitrogen-doped PC fibers derived from cobalt alginate with egg-box structure, which led into the formation of abundant large mesopores, and the product presented an excellent capacitive behavior of 197 F/g at 1 A/g and superb cycling ability (Tang et al., 2018). Geng (Geng et al., 2016) reported a facile method to synthesis HPC derived from sodium alginate by pre-carbonization and NaOH activation, and it exhibited a capacity of 451 F/g in 2 M KOH solution; while its rate performance, which greatly depends on the electrical conductivity of the electrode materials, was not satisfied.

### Starch-Derived PC

**Figure 6:**
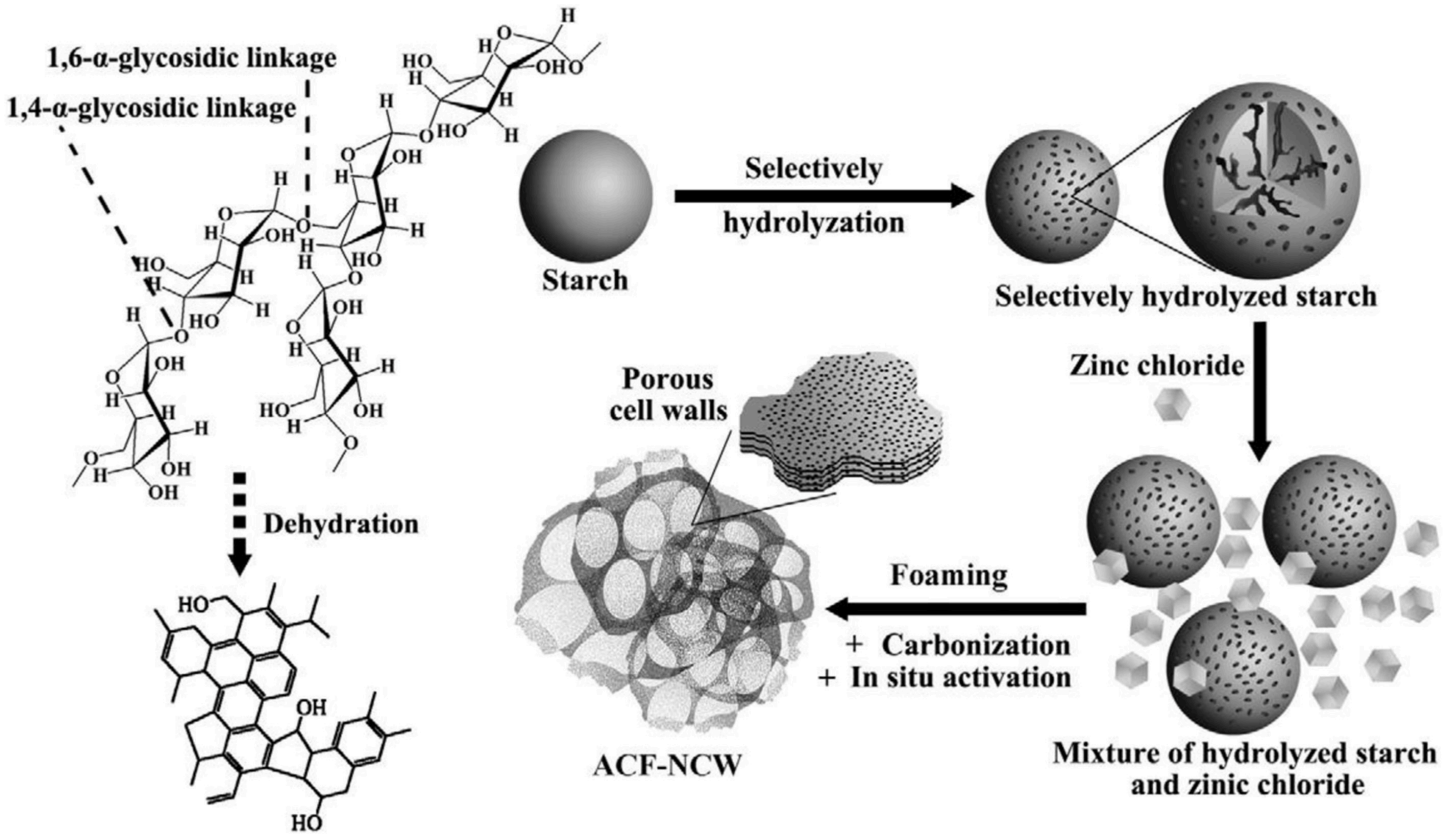
Schematic illustration of the synthesis process of PC foam. Reprinted with permission from Lei et al. (2016). Copyright 2015 Elsevier.

As the most common carbohydrate in the human diet, starch, a polymeric carbohydrate consisting of glucose units jointed by glycosidic bonds, is a promising candidate for supercapacitors as its low cost, accessibility (Lei et al., 2016; Figure 6). Direct carbonization of starch with the aid of activated agents is easy and commonly used by researchers for preparing PC. Guo (Guo et al., 2018) synthesized starch-derived PC through one-pot carbonization, the as-prepared PC achieved a C_s_ of 385 F/g at 1 A/g. Zhang (Zhang et al., 2015b) proposed a simple strategy to fabricate hierarchical hollow porous spherical carbon using starch as raw materials and KHCO_3_ as the activation agent. The relatively large SSA and rich porous structure endowed the electrode materials with a high capacitance (265.4 F/g at 1 A/g) and excellent rate capacitance (137 F/g at 100 A/g). Hence, carbon derived by different botanical origin would influence the electrochemical performance of the products, and this was verified by Bakierska (Bakierska et al., 2017). In addition, hydrothermal treatment of starch is useful for improving the hydrophilic of carbon materials by introducing the oxygen-containing functional group. Therefore, before carbonization and activation at elevated temperature, hydrothermal carbonization was performed, the potato-derived PC spheres displayed a high C_s_ (245 F/g) and good rate performance (61% capacitance retention at 10 A/g) (Qiang et al., 2015); similar processing procedure was used by Hong with sweet potato starch as the carbon source, a C_s_ of 208 F/g at 1 A/g was gained (Hong et al., 2018).

The foaming process (Wang et al., 2016b) through physical or chemical blowing with the aid of carbonate of urea leads to the formation of abundant macropores. For this reason, inspired by bread leavening, Deng (Deng et al., 2015) proposed a non-casting and template-free method to fabricate HPC by mixing the starch with KHCO_3_ followed by carbonization at a high-temperature condition. The as-prepared samples owned a C_s_ of 253 F/g and with no distict capacitance loss after 10,000 cycles. At the same time, Chang (Chang et al., 2017) employed a chemical blowing strategy to synthesis N-doping sheet-like PC by graphitization and chemical activation. The samples gained extremely high surface area (2,129 m^2^/g), large pore volume (0.97 cm^3^/g), good C_s_ (337 F/g at 0.5 A/g) and reasonable cycle stability. Similar preparation strategies were employed to treat starch by other researchers to investigate the electrochemical properties of the PC (Pang et al., 2016a; Yang et al., 2016; Du et al., 2017). Calcium acetate was used as the hard template and mixed with starch before carbonization, the PC with tunable pore size by adjusting the ratio of starch/calcium acetate was prepared. When the current density increased from 0.1 A/g to 10 A/g, the C_s_ of the electrode materials transformed from 277 F/g to 182 F/g, and the cycling results indicated that the PC owned an extremely outstanding cycle stability even after 20,000 cycles (Zhang et al., 2016b). Compared with other kinds of biomass, corn starch is a relatively pure resource as its high-yield character. PC derived corn starch by hydrothermal carbonization, and activation acquired high SSA (1,239 cm^2^/g), good capacitance (144 F/g) and energy density (19.9 Wh/kg), that were performed slightly better than commercial PC (Pang et al., 2016b). The rate performance of the electrode can be improved by combining PC and carbon cloth together. Zhong (Zhong et al., 2018) synthesized a binder-free activated carbon electrode via sol-gel and KOH activation; the corresponding electrodes achieved 272 F/g (1 A/g) and C_s_ retention of 75.9% at 50 A/g.

### Chitin-Derived PC

As one of the most abundant natural polymers, chitins, with stiff chain conformation and considerable nitrogen concentration (~6.9 wt%), can be completely dissolved into the mixture solution of NaOH and urea. Generally, the addition of PETF hampers the electron transport and restrains the rate capacity of electrode materials. Zhang (Zhang et al., 2016a) employed a one-step synthesis strategy for preparing a binder-free PC electrode by sol-gel method. After KOH activation, the electrode achieved gravimetric C_s_ of 272 F/g and 75.9% C_s_ retention at 50 A/g. By means of emulsification and carbonization at high temperature, N-doped microsphere with ample interconnected porous structure, high SSA, unique elasticity and outstanding rate performance were obtained (Suhas and Carrott, 2007). Gao employed prawn shells as a carbon precursor, the obtained N-doped activated carbon exhibited very fine C_s_ of 357 F/g (6 M KOH) and 695 F/g (1 M H_2_SO_4_) (Zhang et al., 2015b). The fungus was also used as PC sources, Long (Ruiz-Rosas et al., 2014) found the corresponding graphene-like carbon possessed high specific surface are (1,103 m^2^/g), outstanding volumetric (360 F/cm^3^) and cycle stability (99% capacitance retention after 10,000 cycles). At the same time, N-doped PC derived from shrimp shells with high surface area (1,271 m^2^/g) by KOH activation also got a high C_s_ (239 F/g at 0.5 A/g). Moreover, as a renewable biomass mainly composed of chitin, cicada slough derived PC was obtained through carbonization in air and KOH etching in inert atmosphere, results showed that the products exhibited fairly high oxygen content (~30%), moderate nitrogen content (~4%) and high C_s_ (266 F/g at 0.5 A/g) (Hu et al., 2014).

### Gelatin-Derived PC

As a renewable biomass resource, gelatin owns abundant rich –NH_3_ groups, which ensure a good wettability after carbonization, and facilitates nitrogen atoms doped during the activation process. Hence, it is believed to be a promising candidate for the raw material of PC for supercapacitor. Recently, it is still a big challenge for preparing bio-sources derived carbon materials with 2D planar architectures, which promote ions transportation and electrons conduction, and further affecting the capacitance and rate performance of the electrode materials. Therefore, utilizing gelatin and dopamine as carbon and N sources, respectively, Fan and colleagues (Fan et al., 2015) developed a general strategy for synthesizing 2D PC nanosheets through a series of treatment, including intercalation, thermal treatment and chemical etching; meanwhile, layered montmorillonite was added to control the microstructure of gelatin, and brought about the formation of two-dimensional nanosheet-like PC. The final products exhibited an enhanced rate capability and a high C_s_ of 246 F/g; besides, the decent capacitance retention (81% at 100 A/g) indicates the electrode material is a good candidate of electrode material for supercapacitor. Furthermore, a free-standing N-doped PC film derived from gelatin/copper hydroxide nanostrands composite films were synthesized by Hu et al. (2016); the binder-free mesoporous N-doped carbon film possessed promoted specific energy (28.1 Wh/kg) and high specific capacity (316 F/g at 0.5 A/g), while its rate performance was not satisfied. Doping with multi-elements is another strategy to improve the electrochemical performance of PC because of the synergistic effect. Therefore, a facile yet sustainable approach was used to produce B/N co-doped PC. Boric acid was added into the gelatin solution, and it acted as hard templates after crystallization during the evaporation of aqueous solution at an elevated temperature. The plate-like shaped boric acid regulating the assembly of gelatin, after carbonization and activation, 2D plate-like PC formed. As the voltage windows is 0.8 and 1.0 V, the C_s_ of the PC is 240 and 230 F/g, respectively. It also showed improved capacitance retention (Zheng et al., 2016b). Except for boric acid, graphene oxide was applied as a regulator to modify the pore structure, composition, and microstructure of PC derived from gelatin, and results showed that the carbon nanosheets with thickness range from 10 to 30 nm, owned high C_s_, decent rate capability and high capacitance retention (76% at 20 A/g). At the same time, Zhang applied a similar approach to preparing layer-like PC with the thickness ~100 nm, which delivered a high discharge C_s_ (366 F/g at 1 A/g), good rate capability (221 F/g at 30 A/g) and suitable cycling performance (Zhang et al., 2015c).

### Plant-Tissue-Derived PC

**Figure 7:**
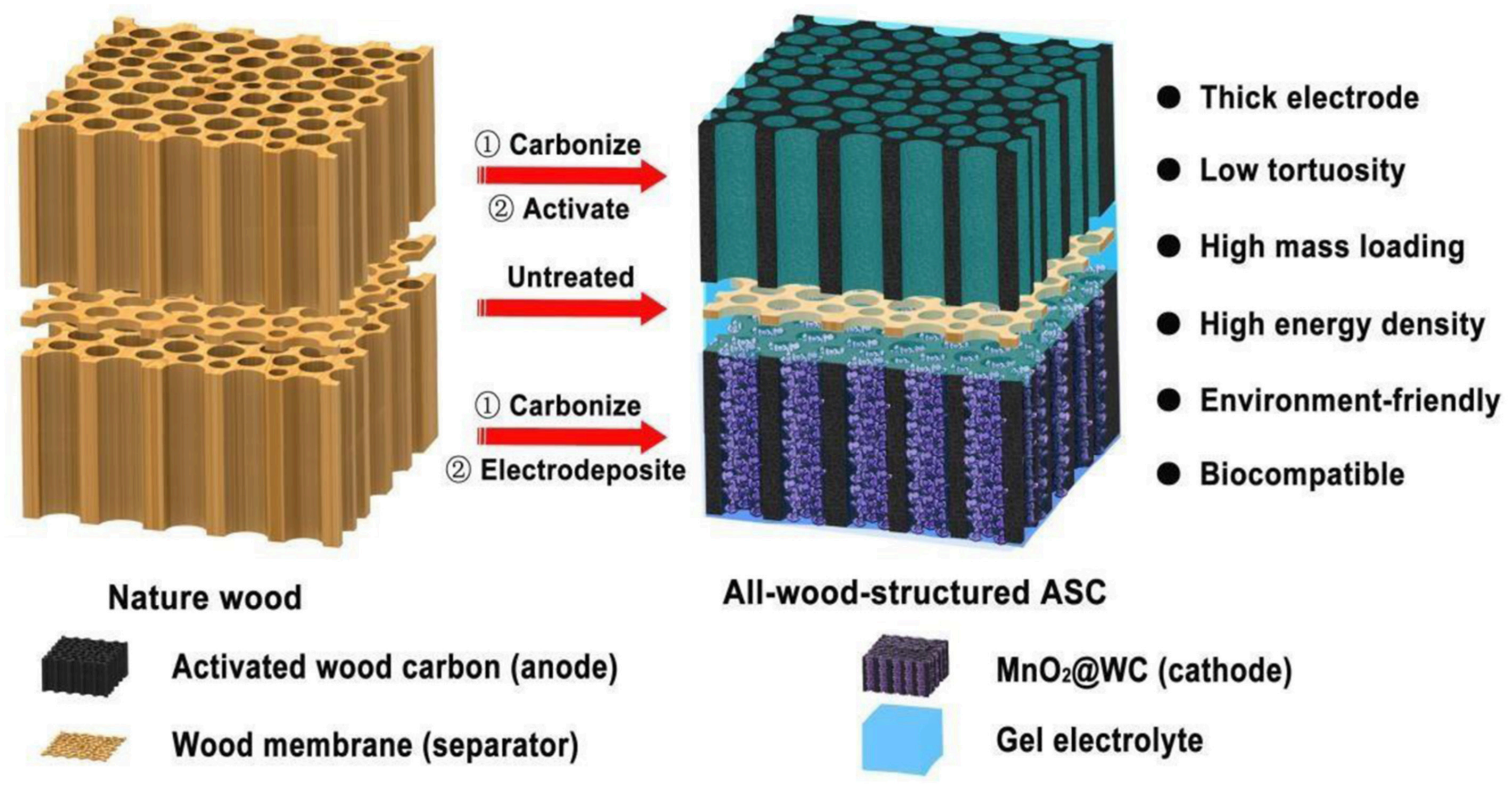
Schematic illustration of hierarchical PC supercapacitor. Reprinted with permission from Chen et al. (2017a). Copyright 2017 Royal Society of Chemistry.

It is well-known that a thick electrode with high areal active materials loading is urgent in supercapacitor designs as it is closely related to the energy density of supercapacitor devices, and it is useful to reduce the cost of manufacturing through maximizing the packing density of electrode materials and decreasing the layers of inactive materials. What is noteworthy is that the transportation rates of the ion and electron are inversely proportional to the thickness of the electrode in the real-world application. However, natural wood possesses a unique anisotropic structure and plenty of open channel along the growth direction, which facilitates alleviating the electrode materials' tortuosity, promoting the ions transfer, and further increasing the rate capacity of the electrode materials (Figure 7). Hence, a surface modified porous wood carbon derived from poplar wood was prepared by Liu (Liu et al., 2012), and the products achieved a maximum gravimetric and volumetric capacitance of 234 F/g and 36 F/cm^3^, respectively. Basswood was carbonized by multistep thermal treatment and CO_2_ activation; the obtained HPC with extremely high mass loading of MnO2 gained high-energy density (1.6 mWh/cm^2^) and power density (24 W/cm^2^) (Chen et al., 2017a). In addition, highly anisotropic, multichannel wood carbon doped by N and S were also exhibited well electrochemical properties, the PC showed a C_s_ of 704 F/g at 0.2 A/g, and still maintained a competitive C_s_ of 349 F/g at 4 A/g (Tang et al., 2018). Pinecone tree activated carbon was prepared by KOH activation at 800°C, and the products showed a C_s_ and energy density of 69 F/g at 0.5 A/g and 24.6 Wh/kg, respectively, besides, it exhibited excellent voltage stability after holding 110 h (Barzegar et al., 2017).

**Figure 8:**
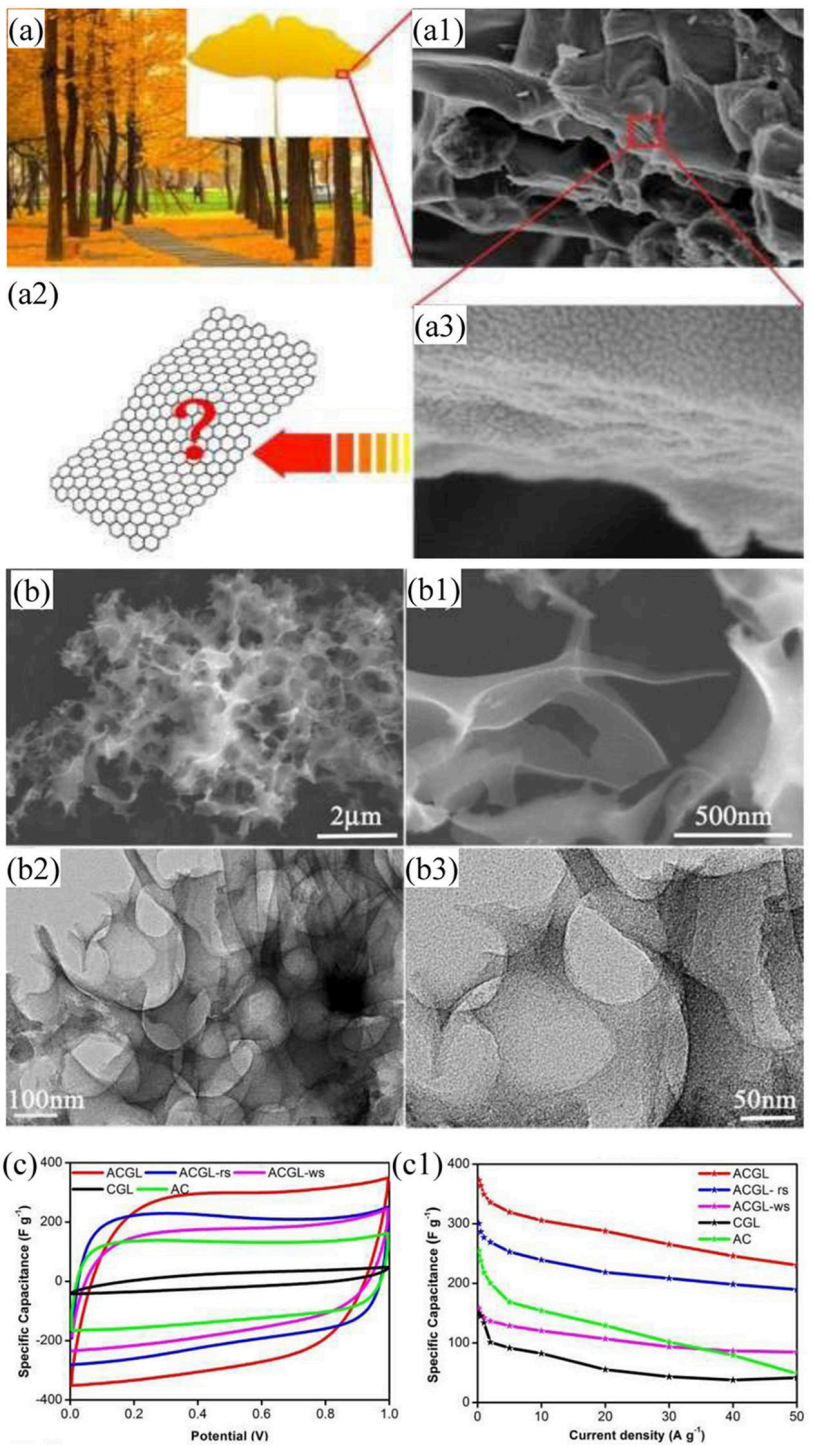
Optical image **(a)** and SEM micrograph of ginkgo biloba **(a1–a3)**; **(b–b3)** SEM and TEM images of carbonized ginkgo biloba; **(c1–c2)** electrochemical performance of PC. Reprinted with permission from Hao et al. (2016). Copyright 2016 Royal Society of Chemistry.

Similar to the wood's microstructure, bamboo-derived carbon was obtained by carbonization with the aid of KOH under N_2_ atmosphere. After being doped by boron and nitrogen, the corresponding HPC exhibited a C_s_ of 281 F/g (1 M, KOH) and energy density (37.8 Wh/kg) (Chen et al., 2015). As N-enrich biomass, the bamboo shoot was carbonized by Chen (Chen et al., 2017c) to prepare N-doped PC materials, which was endowed with high SSA, highly interconnected pores and uniform nitrogen dopant distribution. Its outstanding C_s_ and rate performance ensured it is a promising candidate for supercapacitors. Wei employed broussonetia papyrifera as a biomass source, after hydrothermal treatment in KOH solution, then pyrolysis and activated at high temperature in Ar atmosphere, N-doped HPC with high SSA (1,212 m^2^/g) and outstanding C_s_ (320 F/g at 0.5 A/g) were fabricated (Wei et al., 2015). Thubsuang reported that rubberwood waste treated with H_3_PO_4_ or NaOH led into the formation of carbon monoliths, which exhibited a maximum gravimetric capacitance, volumetric capacitance and energy density of 129 F/g, 104 F/cm^3^, and 14.2 Wh/kg, respectively (Thubsuang et al., 2017). What's more, wood waste was used as raw material, and the N-doped PC-PANI composite possessed a decent C_s_ (347 F/g at 2 A/g) and high energy density (44.4 Wh/kg) (Yu et al., 2015). Compared with conventional KOH activation, impregnate-activation method resulted in PC derived from pine tree sawdust with higher surface area and richer interconnected pores, and achieved good C_s_ in the organic electrolyte (146 F/g) and IL electrolyte (224 F/g) (Wang et al., 2017). Except for these woody materials aforementioned, other woody wastes, such as spruce bark (Sun et al., 2018), Java Kapok tree (Kumar et al., 2018), plane tree (Yao et al., 2017), Melia azedarach (Morenocastilla et al., 2017), auriculiformis tree bark (Momodu et al., 2017), ginkgo leaves (Hao et al., 2017) (Figure 8) were developed as carbon for fabricating PC, whose C_s_ was all better than commercial activated carbon. Musa basjoo, which appears like a tree and have three layers of structure involves nano-/micro-/millimeter level, that endowed the PC with abundant interconnected pores after KOH activation. The products displayed high C_s_ and good cycling performance (Zheng et al., 2016a).

In addition to wood, plants with a similar structure like shrimp shell, which preserve its layered structure after carbonization and washing with an acid solution, should be a kind of promising candidate for HPC. The microstructure of these plants, such as the flower petals andtree leaves, could be kept by shape fixing via a salt recrystallization strategy, which involves the addition of salt crystals with a high melting point (Ding et al., 2015). Hence, the salt sealing strategy was utilized to fabricate graphene-like PC nanosheets (GPCN) derived from salvia splendens' flower petals. The obtained PC consisted of 10.26% O and 2.3% N through elemental analysis, and it owns high SSA (~1,051 m^2^/g). Thanks to these characteristics, the GPCN-based carbon materials exhibited ~220 F/g at 20 A/g, and showed a high-rate capability (86.3% capacity retention from 1 to 100 A/g in 6 M KOH solution) (Liu et al., 2018b). In addition, PC derived from paulownia flower by pyrolysis carbonization and chemical activation also showed high SSA, suitable size distribution, superb wettability and partial graphitization phase, which endowed the electrode with a C_s_ of 297 F/g at 1 A/g in 1 M H_2_SO_4_ solution, and the corresponding electrode material exhibited high-energy densities of ~22 Wh/kg as the power output is 3,781 W/kg (Chang et al., 2015). Moreover, other kinds of flower-derived carbon have been prepared by carbonization and activation process, such as camellia and pine cone flower (Nagaraju et al., 2016; Ma et al., 2017), Borassus flabellifer flower (Sivachidambaram et al., 2017), Osmanthu's flower (Zou et al., 2018), cornstalk (Wang et al., 2018b), rice husk (Dong et al., 2018), grape seeds (Guardia et al., 2019), rice straw (Liu et al., 2018d), walnut shell (Wang et al., 2019), wood sawdust (Sevilla et al., 2019) all of these PC electrode materials delivered a relative high C_s_ and good rate capacity owing to their fine microstructure and abundant N, O elements.

What's more, leaves with a delicate hierarchical structure, which promote the diffusion of electrolytes in living organisms, are also a good choice for transferring it into HPC materials. Li proposed a facile strategy to fabricate a free-standing bio-carbon supercapacitor derived from sisal leaves by being carbonized at 1,000°C for two times and by chemical activation afterwards, was removed and freeze-dried; the developed free-standing electrode exhibited a high C_s_ of 204 F/g at 1 A/g and good rate capacity; at the same time, it also delivered a relative steady capacity in an organic electrolyte (Wang et al., 2016c). It is worth noting that the combination of the physical and chemical activation method might bring about unexpected results. Li (Li et al., 2015d) proposed a novel strategy to treat the fallen leaves by the activations of mixed KOH and KHCO_3_. The combination of KOH and KHCO_3_ led to the enlargement of pore size, which benefited the diffusion of ions and is helpful to enhance the specific capacity and rate performance of carbon materials, the electrochemical characterizations' results revealed that this novel strategy endows the PC with a capacity of 242 F/g at 0.3 A/g in 6 M KOH solution, and it also showed a relative good cyclic performance. Although the activation process is a common method to fabricate biomass-derived PC materials, while the physical or chemical activates, reaction always accompanied with the collapse of the fine microstructure of the biomass, and further affecting the ion/electron diffusion during energy storage. Hence, a possible way should be developed to replace the activation process while endows the PC with high SSA and abundant pore structure. Huang (Huang et al., 2017) proposed a facile technique, which involves indicalamus leaves and polytetrafluoroethylene as carbon precursor and silica-*in-situ*-remover, respectively, the obtained PC owns a SSA of ~1,800 m^2^/g, and exhibited high capacitance (326 F/g in 1 V supercapacitor), high-energy density (23.7 Wh/kg at power density of 224.5 W/kg). Furthermore, hydrothermal treatment is a benefit to introducing rich oxygen-group into the framework of carbon, and dual-doped by N, S can promote the electron conduction and increase the active sites of PC materials. Hao and colleagues reported that HPC derived from gingko leaves by hydrothermal treatment in H_2_SO_4_ and then activated by KOH at elevated temperature in inert atmosphere, the final products contained small amount of N and S according to the EDS analysis; and the PC maintained a high C_s_ of 364 F/g at 0.5 A/g and excellent cycling capability (98% capacity retention after 30,000 cycles) (Hao et al., 2016). In addition the leaves aforementioned, other leaves, such as tea (Inal et al., 2015; Ma et al., 2017), euonymus japonicas (Zhu et al., 2015), corn (Yang et al., 2017), cabbage (Wang et al., 2016c), have been used as carbon precursors for synthesizing PC materials for supercapacitor electrodes.

## Conclusion and Prospective

In the new era of energy storage, compared with traditional activated carbon materials, biomass-derived HPC has achieved superior performance because of their natural fine structure and rich race elements in the organic tissue. However, it is worth noting that their rate capability performance is not satisfied especially at a high charge/discharge electric current condition. That should be contributed to the high concentration of oxygen groups in carbon framework, which is helpful for enhancing the hydrophilic of carbon materials, and further facilitating the infiltration of electrolyte, while these oxygen groups hamper the electric conductivity of PC. Hence, it is urgent to develop an efficient strategy to balance the wettability and electric conductivity of the carbon matrix, and that calls for the joint from theoretical and experimental researches.

Till now, the chemical activation process through KOH, NaOH etc. is a common tool for preparing porous carbon with a rich porous structure, especially for micropores. It should be noteworthy that a large amount of volatile-gases will be formed because of the reaction between KOH and carbon matrix at elevated temperature, these volatile gases, including metal K, CO, CO_2_, H_2_, and H_2_O, not only lead to the formation of abundant micropores, they also threaten the safety of the instrument because of the strong corrosiveness of metal K. Therefore, other activation strategies should be developed to meet the demand of industrialization.

Ion diffusion is a key issue in supercapacitor systems. Although molecular simulation provides different viewpoints for us to design electrode materials with high efficiency; recently, it mainly focuses on the ion-diffusion within ultra-small pores, and it is still a big challenge for us to investigate the diffusion process of ions in hierarchical pore structure, which is extremely important to enhance the electrochemical performance of PC. Moreover, from the perspective of the environment, security and cost, aqueous electrolyte, but not ionic liquids, should be a better choice for the wide application of supercapacitors in energy storage. However, most of the previous studies have been focused on simulating ion behavior in ionic liquids, which is quite different from the aqueous electrolytes, including ion size, solvation shell and diffusion coefficient. Hence, it is necessary to carry out related studies, and investigate the ion diffusion behavior in aqueous electrolytes, then push the development of supercapacitor.

The impact of carbon doping is still unclear, although doped PC materials provide great opportunities to meet the energy density gap between supercapacitor and battery. As we know, introducing nitrogen into the framework of carbon is always accompanied with the enhancement of electrical conductivity and increasing of electrochemically-active sites compared with commercial carbon, which further results in huge improvement of C_s_ because of an additional faradaic contribution, while the enhancement is quite limited in an aqueous electrolyte. Meanwhile, although the promising prospect of aqueous electrolyte, till now, ions liquids offer better capacitive performance, while it still needs to investigate numerous cations, anions and solvent molecules to achieve more excellent performance to meet the requirement of the industry. Therefore, high-throughput techniques, already applied in battery materials and electrolytes, is a powerful tool for the exploitation of promising systems among thousands of candidates for supercapacitors, and the systems-screening process is closely related to the results of *in-situ* experiments and simulation in molecular level.

Unveiling the ion adsorption and charge storage in PC materials for supercapacitor is essential for its applications; in the meantime, it also promotes the development of other energy storage system, for example, redox flow batteries, biofuel cells, flow capacitors. Hence, it is of central importance to combine the experimental and theoretical tools gained from supercapacitors to promote the development of other current/future technologies.

At last, it is noteworthy that previously reported electrode materials with high gravimetric/areal/volumetric capacitance is meaningless if we ignore the corresponding mass loading/working area/ total volume of the electrode. Hence, effective metrics are required to evaluate the performance of numerous materials for supercapacitors, including biomass-derived PC. As for gravimetric capacitance, ultra-small mass loading means the storage of a limited amount of charges. So, the mass loading of electrodes is a key parameter in comparing the capacitance variation, and it is still urgent to improve electrochemical performance of supercapacitor at high mass loading, but not the C_s_ of the electrode with small loading active materials.

## Author Contributions

SY and JZ were responsible for literature searching and drafting. All authors contributed equally to the final writing of the paper.

### Conflict of Interest Statement

The authors declare that the research was conducted in the absence of any commercial or financial relationships that could be construed as a potential conflict of interest.
